# Ketogenic diet ameliorates MASLD via balancing mitochondrial dynamics and improving mitochondrial dysfunction

**DOI:** 10.1038/s41387-025-00391-w

**Published:** 2025-08-25

**Authors:** Yuehua You, Hongbin Ni, Qin Ma, Lincheng Jiang, Jingshu Cai, Wenjun He, Xiaojing Lin, Kemeng Li, Zhuyun Wang, Weiyan Yan, Xiaoqiu Xiao, Li Ma

**Affiliations:** 1 Department of Endocrinology, Sichuan-Chongqing Joint Key Laboratory of Metabolic Vascular Diseases, Chongqing Key Laboratory of Translational Medicine in Major Metabolic Diseases, The First Affiliated Hospital of Chongqing Medical University, Chongqing, China; 2 Department of Nutrition and Food Hygiene, School of Public Health and Management, Chongqing Medical University, Chongqing, 400016 China

**Keywords:** Nutrition, Endocrine system and metabolic diseases

## Abstract

**Background & Aims:**

Ketogenic diet (KD) is recognized as an effective lifestyle intervention for managing metabolic dysfunction-associated steatotic liver disease (MASLD). This research aimed to assess the impact of KD on metabolic parameters in MASLD mice and elucidate the underlying mechanism.

**Methods:**

High-fat diet (HFD)-induced MASLD mice were subjected to KD for 2 weeks. Researchers measured hepatic fat, plasma Alanine Aminotransferase (ALT), and Aspartate Aminotransferase (AST) levels to assess metabolic changes. Hepatic mitochondrial dynamics were examined using transmission electron microscopy and Western blot. Mitochondrial functions were evaluated through Quantitative Polymerase Chain Reaction (qPCR) and measurement of ATP content. In vitro, HepG2 cells were treated with palmitate (PA), β-hydroxybutyric acid (β-OHB), and/or the mitochondrial fusion inhibitor MFI8 to study mitochondrial morphology, function, and lipid deposition.

**Results:**

KD feeding partially improved the MASLD phenotype and reduced Fission 1 protein (Fis1) and Dynamin-related protein 1 (Drp1) levels in the livers of MASLD mice. Additionally, KD ameliorated HFD-stimulated mitochondrial dysfunctions, as evidenced by elevated ATP levels and upregulation of key genes responsible for fatty-acid-oxidation. β-OHB mitigated PA-stimulated mitochondrial dysfunction and fission in HepG2 cells. Furthermore, β-OHB attenuated PA-stimulated lipid deposition, with this effect being counteracted by MFI8.

**Conclusions:**

Our study suggests that a 2-week KD partially alleviates lipid deposition, restores mitochondrial dynamics balance, and improves mitochondrial dysfunctions in the livers of MASLD mice.

## Introduction

Metabolic dysfunction-associated steatotic liver disease (MASLD), previously termed non-alcoholic fatty liver disease (NAFLD), is characterized by an excess of lipids in the liver and components of the metabolic syndrome, such as obesity, type 2 diabetes, hypertension, and dyslipidemia [1]. It’s projected to impact one billion people worldwide, constituting roughly 38% of the entire global populace [2]. In 2024, the FDA approved resmetirom, the first drug for the treatment of non-cirrhotic metabolic dysfunction-associated steatohepatitis [3]. However, resmetirom has side effects such as diarrhea, nausea, and drug-induced hepatotoxicity. Therefore, traditional interventions such as diet and exercise remain healthy, cost-effective interventions for treating MASLD [4]

The ketogenic diet (KD) emphasizes high fats, minimal carbohydrates, and moderate protein [5]. It triggers a metabolic shift from glucose to fatty acids as the primary energy source, leading to the production of ketone bodies for use by other tissues and organs [6]. Besides its well-known role in treating intractable epilepsy, the KD has also been explored for managing various metabolic disorders, including dyslipidemia, insulin resistance, cardiovascular disease risk, and MASLD [7, 8] A meta-analysis has demonstrated that, compared to conventional diets, KD demonstrates greater efficacy in reducing blood lipid, blood glucose, and body weight among overweight individuals, particularly those with type-2-diabetes [9]. KD has also shown promise in improving MASLD phenotypes by aiding in weight loss, enhancing insulin sensitivity, and making ketone bodies potent mediators of fibrosis and inflammation [10, 11]. Another study confirmed that KD reduces HFD-induced hepatic triglyceride levels by balancing mitochondrial homeostasis [12]. However, the key role of mitochondrial-based mechanisms in KD improves MASLD deserves further investigation.

Mitochondria are pivotal in both energy generation and cellular pathways, exhibiting a wide range of functional diversities that are paralleled by their structural and morphological variability throughout the cell cycle [13]. Mitochondrial dynamics are primarily governed by key protein regulators such as Fission 1 protein (Fis1), Dynamin-related protein 1(Drp1), Optic atrophy 1(Opa1), Mitofusin 2 (Mfn2), and Mitofusin 1(Mfn1) [14]. Fis1 and Drp1 facilitate mitochondrial division by constricting and cutting mitochondria, whereas Opa1, Mfn2, and Mfn1 promote mitochondrial fusion by merging mitochondrial membranes. Under normal conditions, mitochondrial fusion and fission are dynamically balanced, but this equilibrium can be influenced by nutrient availability or metabolic demands. For example, KD acts to reduce oxidative stress and control inflammation by increasing mitochondrial efficiency [15]. Pathological syndromes, such as cancer, myopathy, malnutrition, and MASLD, can lead to significant alterations in mitochondrial size. Reductions in liver fatty acid oxidation in MASLD are often accompanied by mitochondrial structural abnormalities [16]. Research suggests that liver-specific knockout of Mfn2 protects mice against high-fat diet-stimulated insulin resistance and obesity [17].

Despite this knowledge, the effect of KD on mitochondrial dynamics and its roles in ameliorating MASLD development are still not well understood. Therefore, in this study, high-fat diet-stimulated MASLD mice were fed a ketogenic diet, and changes in the metabolic phenotype of MASLD were assessed. Furthermore, the roles of mitochondrial dynamics and mitochondrial functions in the ketogenic diet-mediated improvement of MASLD were investigated. Collectively, these findings suggest that the ketogenic diet prevents the metabolic phenotypes of MASLD, restores equilibrium in mitochondrial dynamics, and improves mitochondrial dysfunctions in the livers of MASLD mice.

## Methods

### Animals

Male C57BL/6 J mice (6 weeks old) were procured from Beijing-Vital-River-Laboratory-Animal-Technology-Company. They were maintained in a standard vented cage under controlled temperature and humidity, with a 12-hour light/dark cycle and unlimited access to water. The mice were randomly assigned to one of four groups: standard diet (SD) alone, HFD alone, HFD with KD, and SD with KD (HFD for 12 weeks, KD for 2 weeks). The macronutrient composition of the diets was as follows: SD - 77% carbohydrates, 13% fat, and 10% protein; KD- 90% fat and 10% protein (D12369B, Research Diet, USA); HFD- 30% carbohydrates, 60% fat, and 10% protein (D12492, Research Diet, USA). Each diet was standardized per calorie for micronutrient content, fiber, and preservatives (Supplementary Table 1). Each diet condition consisted of *n* = 5. All mice were weighed, and blood samples were collected following isoflurane anesthesia. Liver samples were obtained following isoflurane euthanasia by cervical dislocation, and their weights were recorded before storing them according to the requirements of different experimental protocols. All experiments were conducted in compliance with the Institutional Animal Care and Use Committees of Chongqing Medical University guidelines (2018-044).

### Cell culture

The HepG2 cell line was sourced from the Shanghai Institute of Cellular Biology, Chinese Academy of Sciences. These cells were cultured in DMEM (Gibco, USA) supplemented with 10% FBS (Biological Industries, Israel) and 1% Penicillin-Streptomycin (Beyotime Biotechnology, China). Upon reaching 70-80% confluence, they were exposed to palmitate (PA) (300 μM; Sigma-Aldrich, USA) and/or β-hydroxybutyric acid (β-OHB) (2 mM; MCE, USA). For PA preparation, PA was dissolved in anhydrous ethanol to create a 300 mM palmitate (PA) solution. This solution was then dissolved in 10% BSA (fatty acid-free, Sigma, USA) to a final concentration of 3 mM, which was mixed with preheated DMEM (BSA/DMEM ratio 1:1, 60 °C, 5 minutes). The HepG2 cells were cultured for 24 hours under the following conditions: Control group (no PA, 75 µmol/L BSA), β-OHB group (no PA, 75 µmol/L BSA, 2 mM β-OHB), PA group (300 μM PA complexed to 75 µmol/L BSA), and PA+β-OHB group (300 μM PA complexed to 75 µmol/L BSA, 2 mM β-OHB).

### Assessment of plasma β-OHB levels

Plasma β-OHB levels were determined using β-OHB-Assay-Kits (Nanjing-Jiancheng-Reagents, Jiangsu, China) following the kit’s protocols.

### Glucose and insulin tolerance tests

One week prior to the test, the mice were subjected to “tail pinching acclimatization” to avoid stress hyperglycemia. For the glucose tolerance test, mice underwent fasting for 14 hours and received glucose (2 g/kg bw) via intraperitoneal injection. Blood was obtained by clipping the end of the mouse tail about 1 mm. Blood glucose levels were assessed using a point-of-care blood glucose monitoring system (Yuwell, Jiangsu, China) at 0/15/30/60/120 minutes after administering glucose. For the insulin tolerance test, mice underwent fasting for 6 hours and received 0.75 U/kg insulin (Recombinant human insulin injection,300 IU/3 mL, Novolin). Blood glucose levels were assessed at 0/15/30/60/90 minutes after injecting insulin.

### Enzyme-linked immunosorbent assay (ELISA)

Hepatic concentrations of IL-1β, TNFα, and IL-6 were assessed using ELISA kits (Jiubang Biotechnology, Quanzhou, China) following the kit’s protocols. Absorbance values were recorded at 450 nm, and hepatic inflammatory factor concentrations were analyzed based on the standard curve.

### Histological staining

Hematoxylin and Eosin (H&E) staining was performed using commercial kits (Solarbio, Beijing, China). Liver tissue was fixed with formalin, dehydrated, and embedded in paraffin for sectioning. Thin sections (3–5 µm thick) were mounted on glass slides, followed by deparaffinization and rehydration. The sections were stained with hematoxylin and eosin. Finally, the slides were dehydrated and mounted with a coverslip. Liver histology scores were determined using the NAFLD Activity Score (NAS) [18]: lobular inflammation (0-3), steatosis (0-3), ballooning degeneration (0-2), and fibrosis scores (0, 1a-c, 2, 3).

Oil Red O staining was performed using commercial kits (Solarbio, Beijing, China). Liver tissue was sectioned as frozen tissue, and the sections were stained with a 0.5% Oil Red O solution in isopropanol for 10-15 minutes at room temperature, washed with 60% isopropanol. After hematoxylin staining of nuclei, hepatic lipid deposition was observed by fluorescence microscope (Zeiss DM4B, Germany).

### Transmission electron microscopy

After fixing overnight in glutaraldehyde (2.5%) at 4 °C, the liver tissues underwent fixation using a 2% osmic acid solution, followed by dehydration through a gradient alcohol series, and embedding in resin. The tissues were then sliced into 70-nm sections using a Leica-EM-UC6-Microsystem, stained with lead citrate and uranyl acetate, and examined using a Philips-CM-120-TEM to visualize mitochondria. Mitochondrial morphology was analyzed using MiNA in ImageJ.

### Western blotting

HepG2 cells and liver tissues were lysed using RIPA buffer (Beyotime-Biotechnology, Shanghai, China) containing PMSF (Beyotime-Biotechnology, Shanghai, China). Following protein content determination using a BCA assay (ThermoFisherScientific, MA, USA), protein specimens were mixed with loading buffer and incubated for 30 minutes at 65 °C. Subsequently, 30 μg of protein specimens were separated on 12% SDS/PAGE gel and transferred onto PVDF membranes (Millipore, MA, USA). After blocking with 5% non-fat milk in TBST, the membranes were incubated overnight at 4 °C with primary antibodies, followed by incubation with secondary antibodies for 60 minutes. The primary antibodies used for immunoblotting included Fis1 (1:1000, 66635-1-Ig, Proteintech, Wuhan, China), Mfn1 (1:1000, A9880, Abclonal, Wuhan, China), Mfn2 (1:1000, 9482, CST, Boston, USA), Drp1 (1:1000, 8570, CST, Boston, USA), and β-actin (1:1000, ZSGB-BIO, Beijing, China). Images were captured and analysis using the Fusion-FX-Spectra-system (Vilber-Lourmat, Paris, France).

### Quantitative Reverse Transcription Polymerase Chain Reaction (qRT-PCR)

Total RNA was isolated from HepG2 cells or liver tissues utilizing RNAiso plus (Takara, Kyoto, Japan), after which cDNA synthesis was conducted using a PrimeScript-RT-reagent-kit (Takara, Kyoto, Japan). Subsequently, RT-PCR was performed with SYBR Green PCR Master Mix (Takara, Kyoto, Japan) in a CFX96-Real-time-System (Bio-Rad, California, USA). Each group was assessed in triplicate. The raw Ct values were normalized to β-actin using the 2^−ΔΔCt^ approach. The primer pairs are provided in Table 1.

**Table 1:** The primer sequences used in this study.

gene	Forward (5’to3’)	Reverse (5’to3’)
Mouse *Cpt1α*	TGGCATCATCACTGGTGTGTT	GTCTAGGGTCCGATTGATCTTTG
Mouse *Pparα*	GGCGTTTCCTGAGACCCT	ATGTTGGATGGATGTGGC
Mouse *Acads*	TTGTGCTGTGAAGTATGCCGA	CTGGTGGCTAATGGCGGTT
Mouse Acadm	CCAGAGAGGAGATTATCCCCG	TACACCCATACGCCAACTCTT
Mouse *slc25a20*	CAACCACCAAGTTTGTCTGGA	CCCTCTCTCATAAGAGTCTTCCG
Mouse *β-actin*	TGGAATCCTGTGGCATCCATGAAAC	TAAAACGCAGCTCAGTAACAGTCCG
Human *Pparα*	GACGTGCTTCCTGCTTCATAGA	CCACCATCGCGACCAGAT
Human *Cpt1α*	CGTCTTTTGGGATCCACGATT	TGTGCTGGATGGTGTCTGTCTC
Human *Acads*	TGCTGGCCTCGATTACCT	GCCTGCTTCTGCTCCTTG
Human *Acadm*	GAGCCATTGATGTGTGCTT	GCTTTTCCTCCGTTGGTT
Human *slc25a20*	CTACCTGTGGCAAGAATGC	CTCCTGGTGATCTGGTTGA
Human *β-actin*	CCTGGCACCCAGCACAAT	GGGCCGGACTCGTCATAC

### Determination of ATP levels

Intracellular ATP content was assessed using an ATP-detection kit (Beyotime-Biotechnology, Shanghai, China). In brief, 30 μg HepG2 cells or liver tissues were lysed in 200 μL lysis buffer. Following centrifugation (12,000 *g*, 5 minutes, 4 °C), the supernatant was utilized to determine ATP concentrations. Then, ATP-detection-working-solution (100 μL) was dispensed into a black 96-well plate. Subsequently, after a 5-minute incubation period, 40 μg supernatant was introduced, and luminescence was quantified using a microplate reader (BioTek-Synergy).

### Evaluation of mitochondrial-membrane-potential (Δψ_**m**_)

HepG2 cells were grown in a 12-well plate and exposed to PA and/or β-OHB for 24 hours. Subsequently, JC-1 dye was used to stain the cells (Beyotime-Biotechnology, Shanghai, China) for 20 minutes at 37 °C. Lastly, the cells underwent three washes with PBS, and the ratio of green-to-red fluorescence was visualized using a fluorescence microscope (ZEISS, Oberkochen, Germany).

### Plasma profiles

Blood specimens were withdrawn from standard diet mice and MASLD mice fed with/without a ketogenic diet. Following centrifugation (3000 rpm, 10 minutes, 4 °C), plasma specimens were utilized to assess liver functions. Both ALT and AST levels were determined using an AU5800-biochemical-analyzer (BeckmanCoulter, California, USA) at the Laboratory Department of the People’s Hospital of Yubei District, Chongqing.

### Measurement of biochemical parameters

Biochemical parameters were assessed using an assay kit (Nanjing-Jiancheng-Reagents, Jiangsu, China). Liver tissues (10 mg) were homogenized in 90 μL ethanol. Following centrifugation (2500 rpm, 10 minutes, 4 °C), hepatic triglyceride (TG) and total cholesterol (TC) concentrations were measured. Total protein content was detected using the BCA approach.

### Immunofluorescence

HepG2 cells were exposed to 300 μM PA and/or 2 mM β-OHB for 24 hours. After fixing with 4% paraformaldehyde for 15 minutes, the cells were rinsed thrice with PBS. Cells were treated with 0.5% Triton X-100 to render them permeable, followed by a blocking step using goat serum. Subsequently, the cells were exposed overnight at 4 °C to primary antibodies targeting Tom20 from Proteintech. On the subsequent day, the cells underwent three washes with PBST before incubation with secondary antibodies for 60 minutes. After 3 additional washes, DAPI was employed for staining the cells, and the image was captured using a confocal fluorescence microscope (ZEISS, Oberkochen, Germany).

### BODIPY and Nile Red staining

HepG2 cells were exposed to PA, β-OHB, and/or MFI8 (20 μM, MCE) for 24 hours. After fixing with paraformaldehyde (4%) for 15 minutes, the cells were stained with either BODIPY or Nile Red dye for 15 minutes, followed by DAPI staining. Images were captured using a fluorescence microscope (Leica, Wetzlar, Germany).

### Statistical analysis

GraphPad Prism v8 was employed for statistical tests. The values are shown as mean ± SEM. Statistical analysis was conducted with One-way ANOVA, followed by Tukey’s post-hoc test. *P* < 0.05 was deemed statistically significant.

## Results

### Ketogenic diet decreases body weight and enhances insulin resistance in MASLD mice

**Fig. 1:**
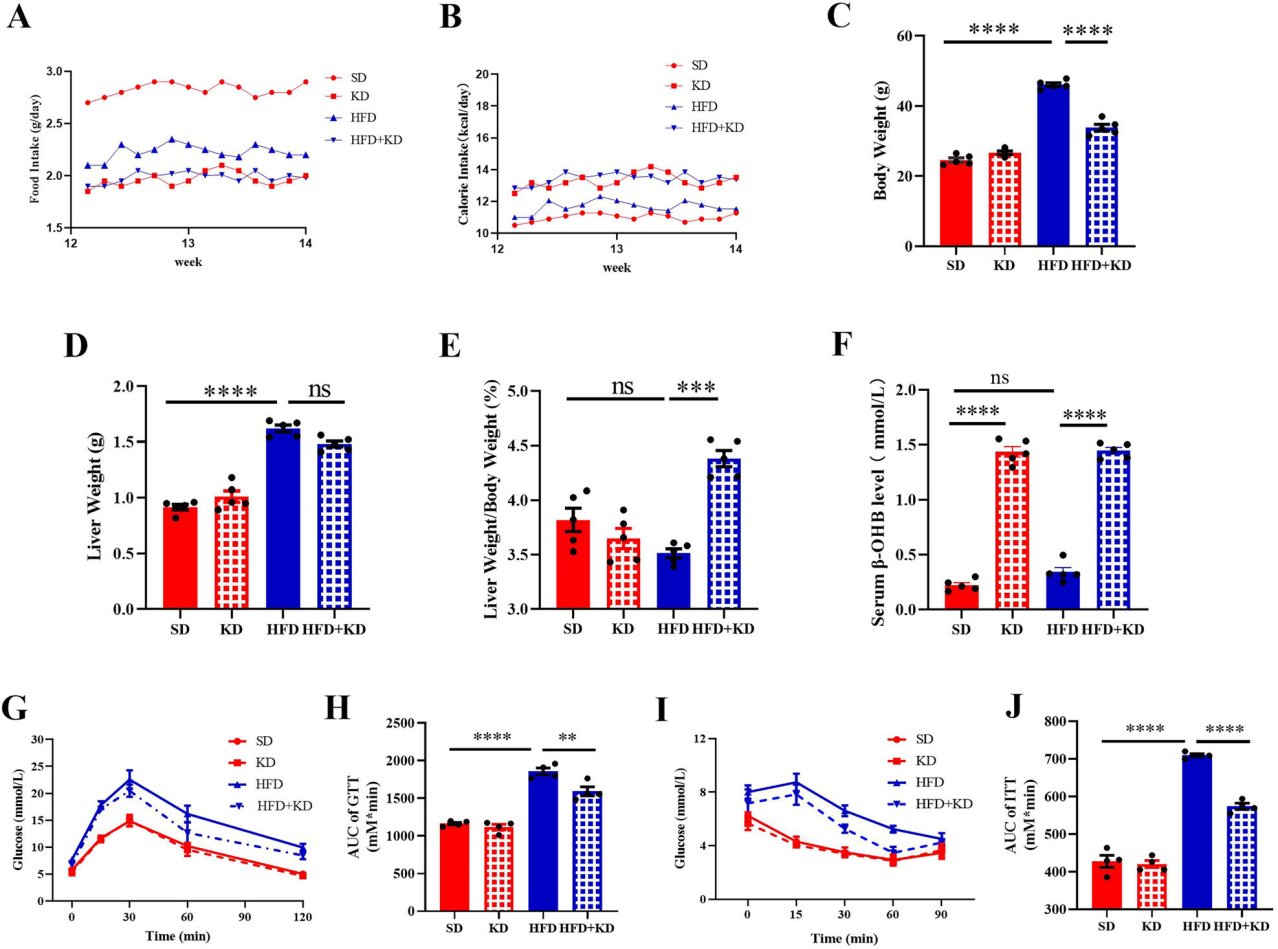
Ketogenic diet decreases body weight and enhances insulin resistance in MASLD mice. **A** Food intake of mice fed with SD, KD, HFD and HFD + KD groups. **B** Calorie intake of mice fed with SD, KD, HFD and KD and HFD + KD. **C** Body weight of different group. **D** Liver weight of different group. **E** The change of the ratio of liver weight and body weight. **F** Plasma ketones β-OHB levels. **G** Glucose tolerance test and AUC (**H**). **I** Insulin tolerance test and AUC (**J**) Data are presented as mean ± SEM. *P < 0.05, **P < 0.01, ***P < 0.001, ****P < 0.0001; n = 5 per group. SD Standard diet, KD Standard diet followed by ketogenic diet, HFD Standard diet followed by high-fat diet; HFD + KD High-fat diet followed by ketogenic diet.

To evaluate the benefits of a ketogenic diet for managing MASLD, we subjected standard diet and MASLD mice to a ketogenic diet for two weeks. Despite a reduction in food intake in ketogenic diet-fed mice compared to those on a high-fat diet (Fig. 1A), the calorie intake (kcal) remained similar between the ketogenic diet and high-fat diet groups (Fig. 1B). As depicted in Fig. 1C–E, the ketogenic diet remarkably decreased both body weight and liver weight in MASLD mice compared to those not on the diet. Predictably, the ketogenic diet also led to an increase in serum β-OHB levels (Fig. 1F). Furthermore, the ketogenic diet could improve glucose tolerance (Fig. 1G, H) and insulin resistance (Fig. 1I, J) in MASLD mice. These data suggest that the ketogenic diet is effective in decreasing body weight and managing blood glucose levels in MASLD mice.

### Ketogenic diet alleviates high-fat diet-triggered steatosis

**Fig. 2:**
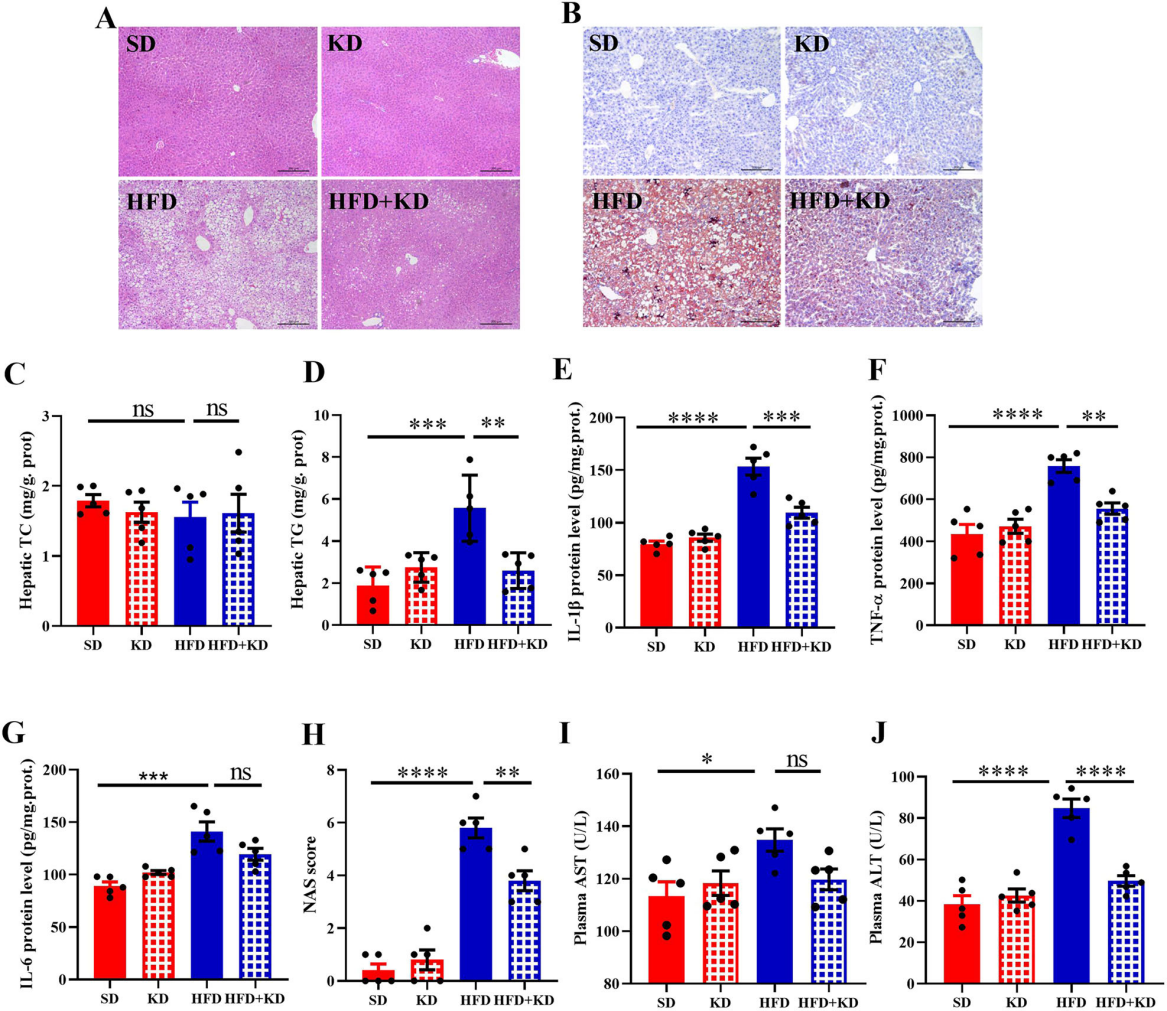
Ketogenic diet alleviates high-fat diet-triggered steatosis. **A** Mouse hepatic staining by H&E and Oil red O staining (**B**). **C**, **D** Hepatic TC and TG measurements. Hepatic cytokine IL-1β (**E**), TNF-a (**F**) and IL-6 (**G**) were measured by ELISA. **H** Hepatic NAS score. **I**, **J** Plasma AST and ALT measurements. Data are presented as mean ± SEM. *P < 0.05, **P < 0.01, ***P < 0.001; *n* = 5 per group. ALT alanine aminotransferase, AST aspartate transaminase, TG Triglyceride, TC Total cholesterol, IL-1β Interleukin 1 β, TNFα Tumor Necrosis Factor α, IL-6 interleukin 6.

To determine the effect of ketogenic diet on hepatic lipid deposition in MASLD mice, hepatic lipid content was examined using H&E and Oil-Red-O staining (Fig. 2A, B). These analyses revealed a significant reduction in lipid content in MASLD mice fed with ketogenic diet. Further analysis of hepatic lipid levels showed no change in hepatic TC levels (Fig. 2C), but a significant reduction in hepatic TG levels in MASLD mice after ketogenic diet feeding (Fig. 2D). Regarding inflammatory response, the levels of Interleukin 1 β (IL-1β), Tumor Necrosis Factor α (TNFα), and Interleukin 6 (IL-6) were obviously upregulated in the liver of MASLD mice but were reduced after ketogenic diet feeding (Fig. 2E–G). Additionally, liver NAS scores, which were significantly increased by high-fat diet, decreased following ketogenic diet feeding (Fig. 2H). While plasma AST levels exhibited no significant alterations (Fig. 2I), circulating ALT levels, initially elevated in MASLD mice, were notably diminished following ketogenic diet administration (Fig. 2J). These findings suggest that ketogenic diet intervention results in reduced hepatic lipid deposition in MASLD mice.

### Ketogenic diet prevents excessive mitochondrial fission in the liver of MASLD mice

**Fig. 3:**
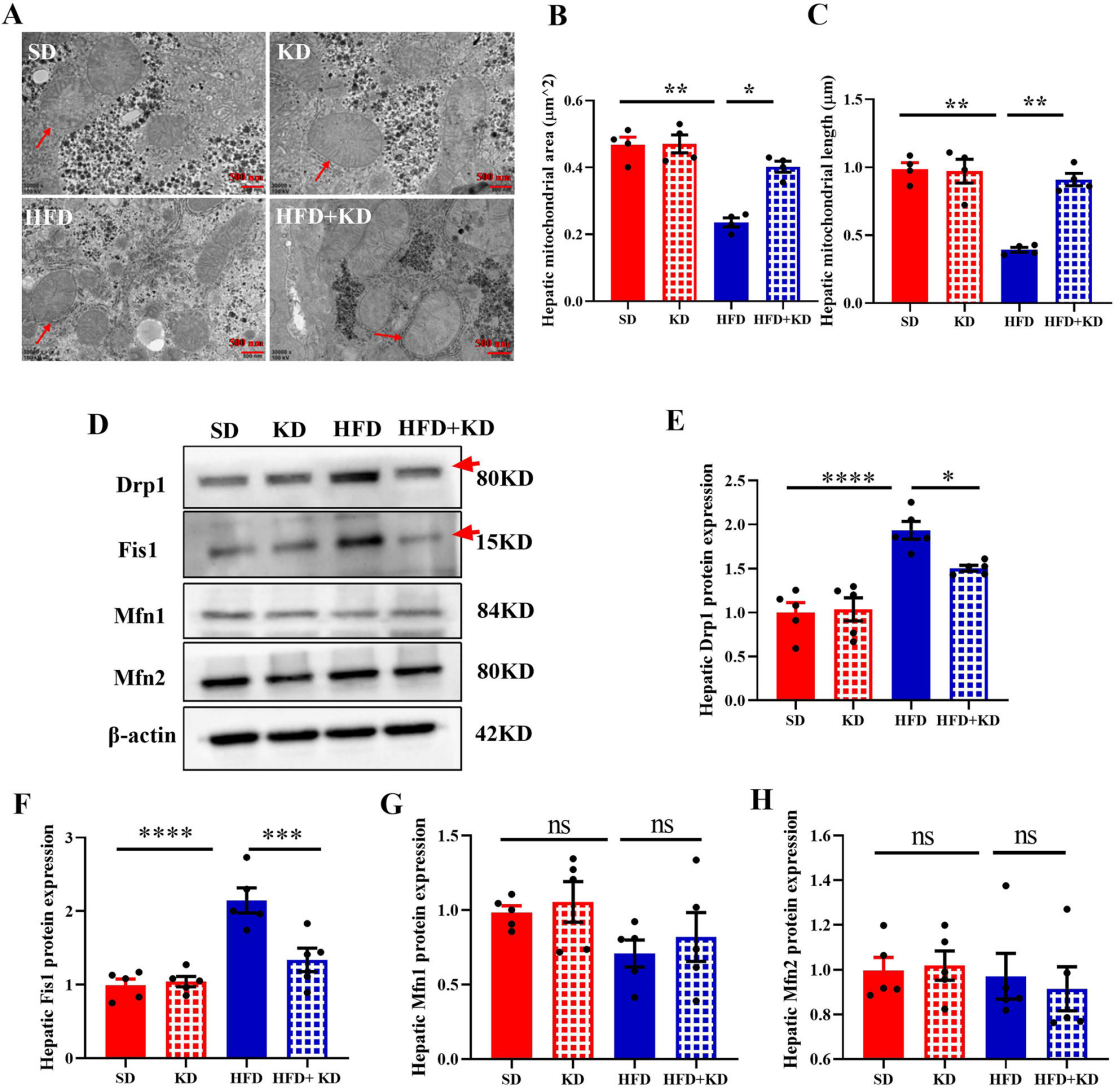
Ketogenic diet prevents excessive mitochondrial fission in the liver of MASLD mice. **A** Mitochondrial morphology was observed by transmission electron microscopy. **B** Mitochondrial area and mitochondrial length (**C**) of SD mice and MASLD mice after feeding with/without KD for 2 weeks. **D** Western blot and quantification analysis of Drp1 (**E**), Fis1(**F**), Mfn1 (**G**) and Mfn2 (**H**) protein levels in the liver of SD and MASLD mice after feeding with/without KD for 2 weeks. Data are presented as mean ± SEM. *P < 0.05, ***P < 0.001, ****P < 0.0001; *n* = 4 for **A**–**C**. *n* = 5 for **D**–H. Mitochondrial length and area were measured according to the scale in image J software. Fis1 Fission 1 protein, Drp1 Dynamin-related protein 1, Opa1 Optic atrophy 1, Mfn2 Mitofusin 2, Mfn1 Mitofusin 1.

Given the key role of mitochondria in lipid metabolism, this study examined whether a ketogenic diet affects mitochondrial morphologies in the livers of MASLD mice. As anticipated, a high-fat diet caused hepatic mitochondrial fission, as indicated by a decrease in mitochondrial size in MASLD mice (Fig. 3A–C). To explore the underlying mechanisms of ketogenic diet on mitochondrial dynamics, the levels of key proteins related to mitochondrial dynamics were detected (Fig. 3D). Both Fis1 and Drp1, which enhance mitochondrial fission, had increased protein levels in the livers of MASLD mice and decreased after feeding a ketogenic diet (Fig. 3E, F). There were no significant differences in the levels of Mfn1 and Mfn2 (Fig. 3G, H). These findings suggest that ketogenic diet feeding prevents excessive liver mitochondrial fission in MASLD mice by mediating mitochondrial dynamics-associated proteins.

### β-OHB prevents PA-stimulated excessive mitochondrial fission in HepG2 cells

**Fig. 4:**
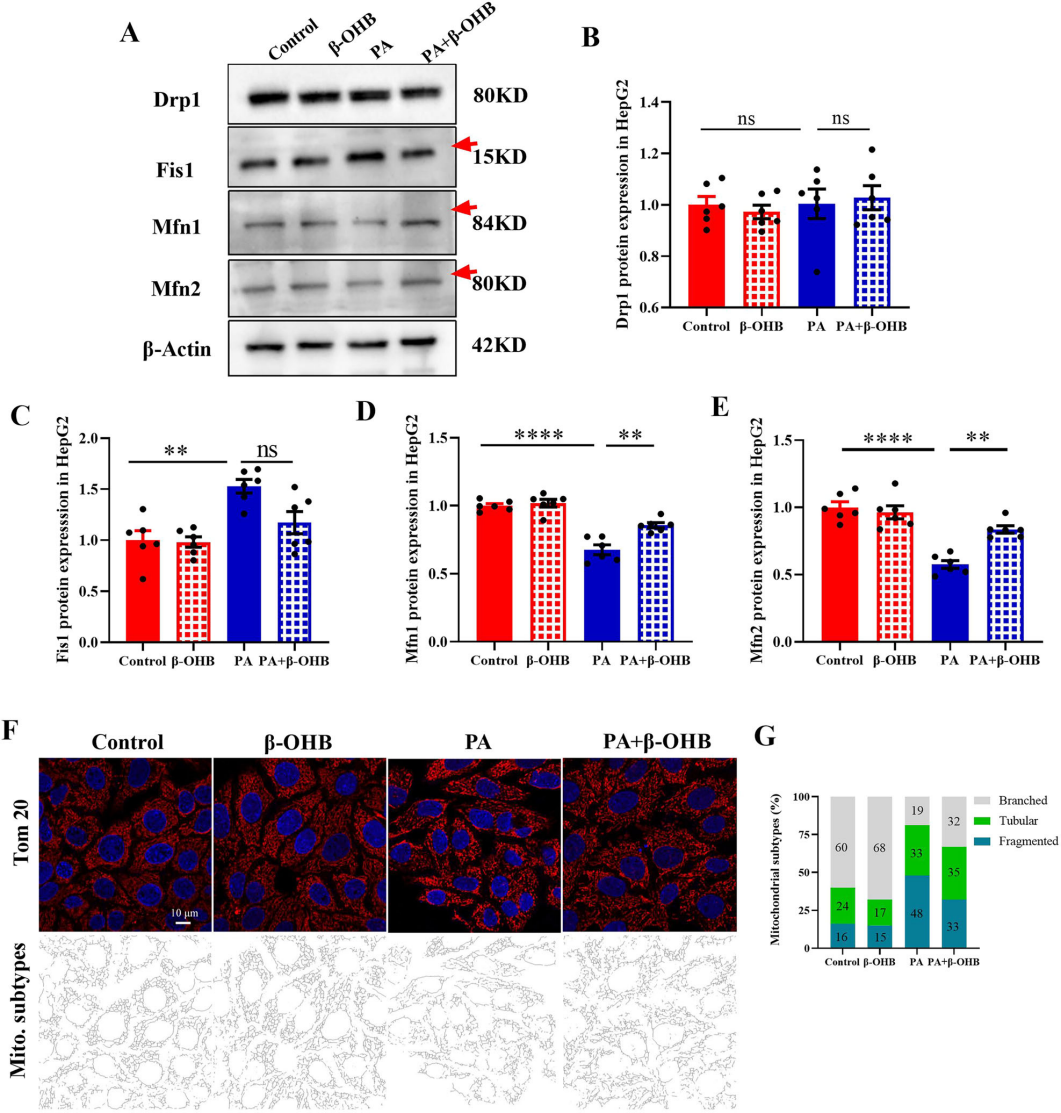
β-OHB prevents PA-stimulated excessive mitochondrial fission in HepG2 cells. **A** Western blot and quantification analysis of Drp1 (**B**), Fis1 (**C**), Mfn1 (**D**) and Mfn2 (**E**) protein levels in HepG2 cells that were treated with PA or/and β-OHB for 24 h. **F** Immunofluorescence staining of Tom 20 and mitochondrial subtypes analysis (**G**) in HepG2 cells in response to PA or/and β-OHB. Data are presented as mean ± SEM. *P < 0.05, **P < 0.01, ***P < 0.0010. ****P < 0.0001; n = 6 for **A**–**E** and *n* = 5 for **F**–**G**. Mitochondrial subtypes analyzed through MiNA in ImageJ.

We subsequently examined whether β-OHB influences mitochondrial morphologies in HepG2 cells subjected to PA exposure. PA treatment markedly elevated the protein levels of Fis1 and reduced those of Mfn2 and Mfn1, without altering Drp1 levels (Fig. 4A–E). Interestingly, these alterations were reversed by β-OHB treatment (Fig. 4A–E). Mitochondrial morphology was then evaluated using Tom20 fluorescence staining. Our findings demonstrated that PA treatment elevated and reduced the number of fragmented and branched mitochondria, respectively, compared to control group. Conversely, β-OHB treatment promoted mitochondrial fusion, as indicated by higher and lower branched and fragmented mitochondria, respectively, compared to PA treatment group (Fig. 4F, G).

### Ketogenic diet improves high-fat diet-stimulated mitochondrial dysfunction

**Fig. 5:**
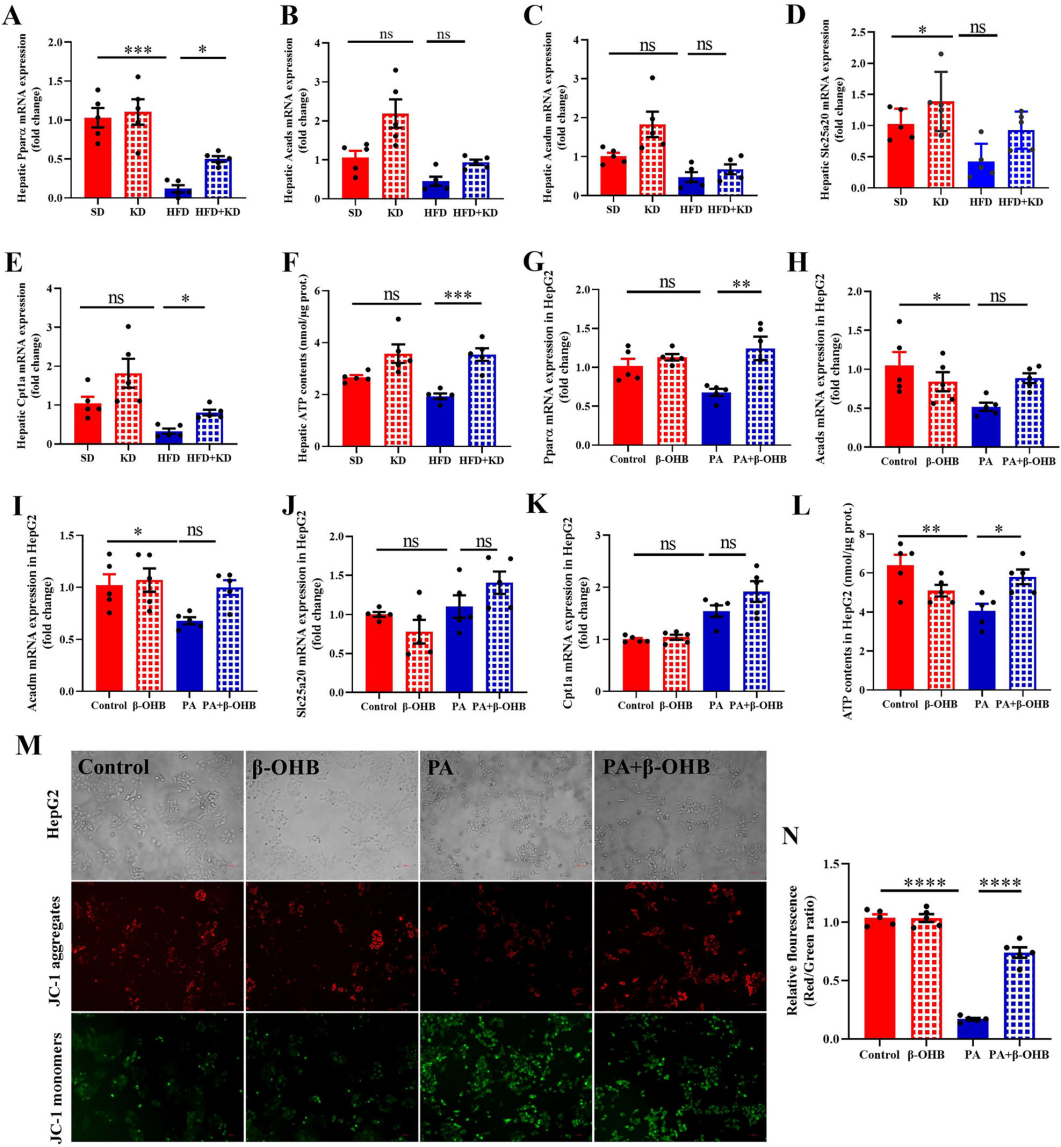
Ketogenic diet improves high-fat diet-stimulated mitochondrial dysfunction. qPCR analyzed the mRNA changes of *Pparα* (**A**), *Acads* (**B**), *Acadm* (**C**), *slc25a20* (**D**), and *Cpt1a* (**E**) in mouse hepatic tissues from SD mice and MASLD mice with/without KD for 2 weeks. **F** Hepatic ATP measurements. qPCR analyzed the mRNA changes of *Pparα* (**G**), *Acads* (**H**), *Acadm* (**I**), *slc25a20* (**J**) and *Cpt1a* (**K**) in HepG2 cells under PA or/and β-OHB treatment. **L** The ATP content in HepG2 cells in response to PA or/and β-OHB. Fluorescence images of JC-1 staining (**M**) in HepG2 cells and relative fluorescence analysis (**N**). Data are presented as mean ± SEM. *P < 0.05, **P < 0.01, ***P < 0.001, n = 5 per group. Cpt1a Carnitine palmitoyltransferase 1A, Slc25a20 Solute carrier family 25 member 20, Acadm Acyl-CoA dehydrogenase, medium-chain, Acads Acyl-CoA dehydrogenase, short-chain, Ppara Peroxisome proliferator-activated receptor alpha.

Mitochondrial morphological abnormalities lead to mitochondrial dysfunction. high-fat diet feeding diminished fatty acid β-oxidation, as indicated by remarkably decreased hepatic mRNA levels of *Carnitine palmitoyltransferase 1* *A* (*Cpt1a*)*, Solute carrier family 25 member 20* (*Slc25a20*)*, Acyl-CoA dehydrogenase, medium-chain (Acadm), Acyl-CoA dehydrogenase, short-chain (Acads)*, and *Peroxisome proliferator-activated receptor alpha* (*Ppara*) in MASLD mice (Fig. 5A–E). However, ketogenic diet partially elevated liver β-oxidation in MASLD mice, reversing the high-fat diet-induced down regulation of *Cpt1a* and *Ppara* (Fig. 5A and E). Moreover, ATP levels in the livers of MASLD mice markedly elevated after ketogenic diet feeding (Fig. 5F). Additionally, the mRNA levels of β-oxidation-associated genes, such as *Cpt1a, Slc25a20, Acadm, Acads*, and *Ppara*, were detected in HepG2 cells (Fig. 5G–K). The effects of PA were restored by β-OHB treatment for *Ppara* (Fig. 5G). However, no obvious changes were noted in the mRNA levels of *Acads*, *Acadm*, *Slc25a20*, and *Cpt1a* after exposure to β-OHB (Fig. 5H–K). However, the decrease in ATP levels triggered by PA was also counteracted by β-OHB (Fig. 5L). Furthermore, the mitochondrial potential difference in HepG2 cells exposed to PA and/or β-OHB was evaluated using JC-1 staining. Fluorescence images indicated that β-OHB ameliorated the PA-stimulated reduction in mitochondrial potential difference (Fig. 5M, N). Collectively, these findings demonstrate that ketogenic diet or β-OHB improves high-fat diet- or PA-stimulated mitochondrial dysfunction.

### β-OHB attenuates PA-stimulated lipid deposition in HepG2 cells

**Fig. 6:**
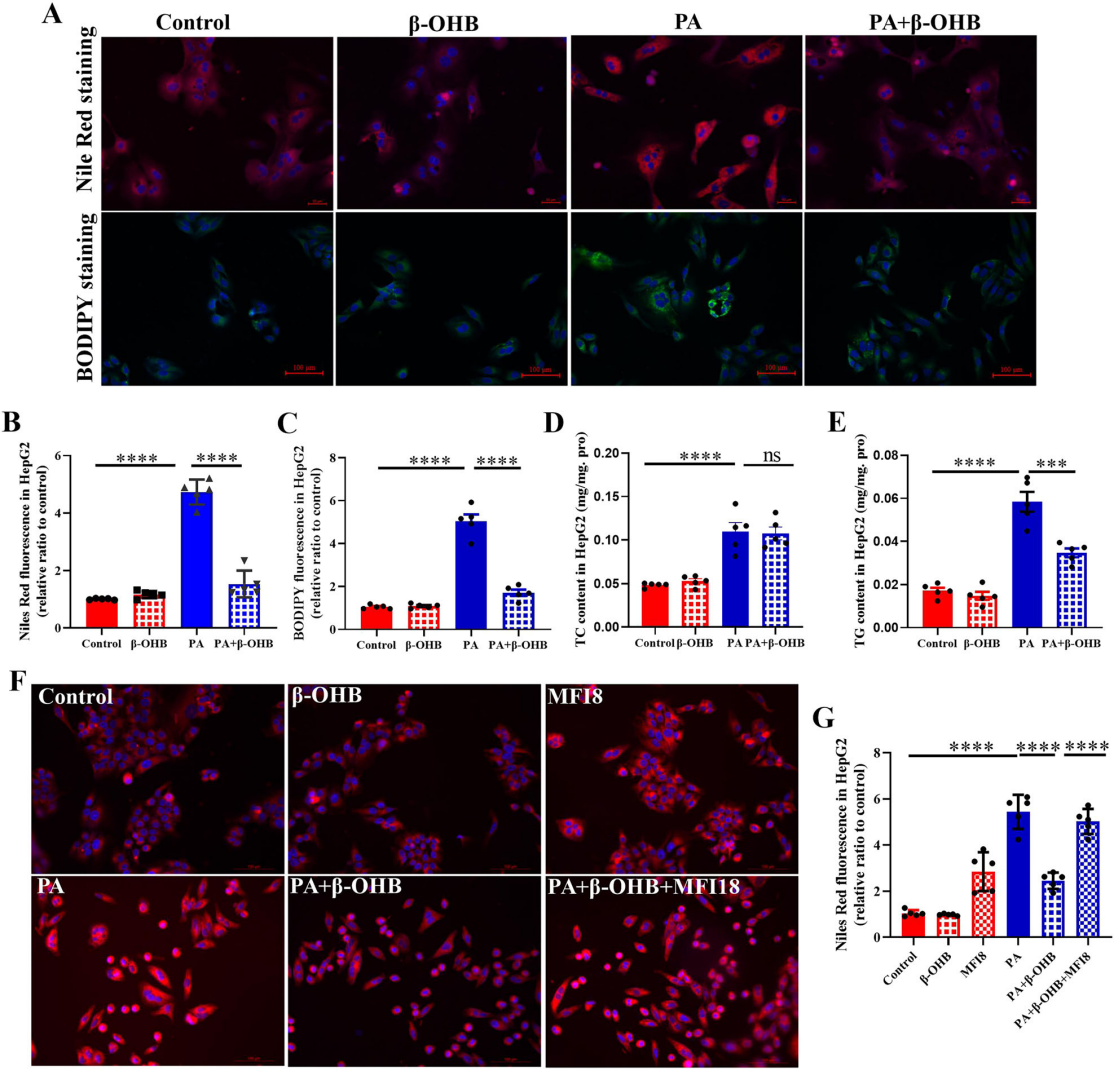
β-OHB attenuates PA-stimulated lipid deposition in HepG2 cells. **A** Nile Red staining and BODIPY staining in HepG2 cells were treated with PA and/ or β-OHB. The relative fluorescence quantification analysis of Nile red staining (**B**) and BODIPY staining (**C**). The TC (**D**) and TG (**E**) content in HepG2 cells. **F** Nile Red staining and relative fluorescence quantification of neutral lipids in HepG2 (**G**) were treated with PA, β-OHB and/or MFI8 for 24 h. Data are presented as mean ± SEM. *P < 0.05, **P < 0.01, ***P < 0.001; n = 5 per group. PA: 300 μM; β-OHB: 2 mM; MFI8: 20 μM.

Considering that serum β-OHB levels rose with ketogenic diet feeding (Fig. 1F), we explored whether β-OHB alleviated PA-stimulated lipid deposition. As depicted in Fig. 6A–C, BODIPY and Nile-Red staining indicated that PA elevated lipid droplets in HepG2 cells, an effect mitigated by β-OHB. Furthermore, PA significantly elevated TC and TG levels (Fig. 6D and E), whereas β-OHB reversed the increase in TG levels. These results suggest that β-OHB reduces PA-induced lipid accumulation. To further examine whether β-OHB improves PA-induced lipid accumulation by influencing mitochondrial dynamics, MFN18 was employed. Notably, MFN18 elevated lipid accumulation and negated the beneficial effect of β-OHB on lipid deposition (Fig. 6F, G).

## Discussion

There have been significant advancements in the understanding of MASLD in recent years, leading to its reclassification as metabolic-associated steatotic liver disease and emphasizing the crucial role of type 2 diabetes in severe MASLD [19]. Disruptions in lipid metabolism are pivotal in the initiation and progression of MASLD [20, 21]. Dietary interventions are among the most effective treatments for MASLD [22]. However, concerns have been raised regarding the safety of the ketogenic diet for treating MASLD due to its potential to increase serum cholesterol levels [23] and impair liver functions [24, 25]. In our prior research, we observed that ketogenic diet might have a temporary beneficial effect on MASLD [26]. Thus, in this research, MASLD mice were fed a ketogenic diet for 14 days, identified as the optimal duration for ketogenic diet to alleviate MASLD. The data showed that a 2-week ketogenic diet significantly reduced the body weight of MASLD mice (Fig. 1A, B). Notably, the impact of ketogenic diet on weight reduction was more pronounced than its effect on liver weight, resulting in an increased liver-to-body weight ratio (Fig. 1C). While the impact of ketogenic diet on liver weight reduction is slightly less than its effect on total weight loss, achieving a 5% weight loss is a significant factor in improving MASLD [27]. Thus, the weight loss effect of ketogenic diet is a key mechanism in the improvement of MASLD.

However, does the source and composition of the triglyceride in a ketogenic diet affect its effects? In a recent study on the effects of ketogenic diets on cellular senescence [28], the authors chose two different ketogenic diet: crisco versus cocoa butter–based (different ratios of saturated versus unsaturated fatty acids), and no difference in body weight and serum ketone levels was observed between the two kinds of ketogenic diets. Our previous studies have also shown that the improvement effect of ketogenic diet on MASLD is related to the liver’s ketogenic capacity, plasma β-OHB levels peaked at 2 weeks after KD feeding, then decreased at 0.8 mmol/L after 6 weeks of KD feeding, which are consistent with the blood biochemical indexes (blood lipids, AST, ALT and etc.) and liver morphological changes in MASLD mice [26]. However, other properties of fat (e.g., types of fatty acids-saturated fatty acids) may have an impact on the effects of the ketogenic diet. Studies have shown that unsaturated fatty acids induce higher ketosis and improve insulin sensitivity without negatively affecting cholesterol levels, suggesting potential benefits of unsaturated fatty acids on hepatic lipid metabolism [29]. In contrast, another study suggests that saturated fatty acids (SFAs) may be strongly associated with mitochondrial dysfunction in NAFLD, with potential mechanisms of overproduction of reactive oxygen species, and disruption of mitochondrial membrane structure [30]. Medium-chain triglycerides (MCT), as a modified version of KD with unique metabolic properties, have been found to deplete hepatic lipid droplets and increase insulin sensitivity in mice [31]. The above composition of fatty acid species may play an important role in the ketogenic diet, especially in physiological processes involving energy metabolism and ketone body production. Future studies need to consider the compositional and structural properties of fats more comprehensively. For example, through techniques such as nuclear magnetic resonance (NMR), the phenomenon of regional isomerization of fatty acids and its influence on the effects of ketogenic diets can be analyzed in more detail. In addition, we plan to further explore the potential differences in the metabolic effects of different fat sources on the ketogenic diet in subsequent studies to more comprehensively assess the contribution of fat composition to the ketogenic diet.

Mitochondria play a crucial role in regulating hepatic lipid metabolism [32]. Under normal conditions, mitochondrial fusion and fission are in a state of dynamic equilibrium. Disruption of this balance can lead to mitochondrial dysfunction and, consequently, various diseases [33]. Numerous studies have shown that the ketogenic diet can enhance mitochondrial morphologies and functions, which benefits conditions such as autism spectrum disorder and mitochondrial diseases [34, 35]. Yet, it is unclear whether the ketogenic diet can enhance mitochondrial morphologies in the livers of MASLD mice, thus contributing to the improvement of MASLD phenotypes. In our MASLD model, we validated that noticeable liver dysfunctions and lipid deposition were associated with excessive mitochondrial fission. Alterations in mitochondrial dynamics proteins contributed to these mitochondrial abnormalities [32]. For instance, in cardiac myocytes, DRP1 inhibition is linked to insufficient mitochondrial respiration, while in skeletal muscle, DRP1 overexpression reduces myofiber oxidative capacity [36]. Interestingly, although knocking down Mfn1 in the liver confers protection to mice against high-fat diet-stimulated insulin resistance and obesity [17], inhibiting the division-related proteins Fis1 and Drp1 in cells impairs cell respiration and insulin secretion [37]. This suggests the importance of maintaining the balance of mitochondrial dynamics. In our study, ketogenic diet feeding inhibited excessive mitochondrial fission, which may be a key mechanism in protecting against MASLD. At the molecular level, our findings revealed an obvious reduction in the levels of Fis1 and Drp1 following ketogenic diet intervention, indicating their potential as targets for further investigation into the management of MASLD.

Disruption of mitochondrial dynamics results in mitochondrial dysfunction [38, 39]. The initial stage in the progression of MASLD involves the excessive accumulation of fat within hepatocytes. Hepatic lipid metabolism encompasses 4 primary pathways: lipid secretion, fatty acid β-oxidation, de novo lipogenesis, and lipid uptake [40, 41]. When the intake and synthesis of fatty acids surpass their oxidation and exportation, hepatic steatosis and MASLD occur [42]. Since mitochondria are the primary sites for de novo lipogenesis and fatty acid oxidation [43], sustaining optimal mitochondrial function is vital for effective lipid metabolism. Furthermore, fatty acid oxidation plays a significant role in the pathophysiology of insulin resistance, obesity, diabetes, heart failure, and kidney fibrosis [44–46]. Our investigation revealed that a ketogenic diet enhanced mitochondrial function, as indicated by elevated fatty acid oxidation and ATP generation in the liver of MASLD mice. Thus, enhancing mitochondrial functions is a key mechanism by which the ketogenic diet protects against MASLD.

As the predominant ketone body in the circulatory system, β-OHB has been reported to aid in mitochondrial repair and improve respiratory functions in endothelial and cardiac cells [47, 48]. Nevertheless, its impact on mitochondrial morphology and function in hepatocytes is not well-documented. Our findings indicate that β-OHB alleviates PA-stimulated mitochondrial fission and dysfunction in HepG2 cells. Interestingly, our findings indicated that without PA, the supplementation of β-OHB suppressed mitochondrial ATP release in HepG2 cells. It is worth noting that the ketogenic process and the tricarboxylic acid cycle represent two key pathways of fatty acid oxidation [49]. However, the impact of β-OHB on the tricarboxylic acid cycle in liver mitochondria under normal physiological conditions remains uncertain. Thus, additional research is needed to elucidate the impact of β-OHB on mitochondrial ATP release in the ketogenic tissue under normal conditions.

In the present study, we focus on β-OHB mainly because it is one of the most predominant ketone bodies in the ketogenic diet, accounting for 78% of the total number of plasma ketone bodies, and has a wide range of metabolic modulatory functions in both physiologic and pathologic states. In addition, acetoacetate has an antioxidant effect and participating in the process of energy metabolism in mitochondria to maintain the normal function and energy metabolism of mitochondria [50]. Acetone is a secondary product formed by the spontaneous decarboxylation of acetoacetic acid, which is metabolized in vivo mainly by exhalation or excretion [51], and has the least metabolic impact compared to other ketone bodies. Therefore, more studies are needed to confirm whether the ketone body type significantly alters MASLD outcomes and mitochondrial function. This complexity emphasizes the need for future studies to elucidate these relationships, particularly in preclinical models.

Finally, the clinical use of the ketogenic diet continues to warrant our attention. The relationship between the ketogenic diet and MASLD has received particular attention over the past few years. In a cohort of 27 obese patients, a 4-week ketogenic diet (71% fat, 25% protein) resulted in a 19.8% decrease in liver volume [52]. Mardinoglu et al. reported a 2-week KD intervention in 17 patients diagnosed with obesity and NAFLD. MRS assessment revealed a significant reduction in hepatic fat content, accompanied by a reduction in hepatic lipogenesis gene expression [53]. In a recent prospective study, which included 33 obese participants who were treated with a very-low-calorie ketogenic diet (VLCKD) for 8 weeks, the fatty liver index, fasting glucose, lipid levels, ALT, and systolic and diastolic blood pressure levels were significantly reduced after VLCKD [54]. These clinical studies suggest that the ketogenic diet has a significant ameliorative effect on NAFLD in the short and medium term, independent of calorie and fat intake. In order to better link our findings to clinical practice, we plan to gradually expand the scope of our study to the clinical level in future studies. This may include conducting prospective cohort studies or randomized controlled trials to assess the direct impact of the ketogenic diet on clinical outcomes (e.g., liver function markers, quality of life improvement, etc.). In addition, we will also focus on the strength of associations between surrogate markers and clinical outcomes to ensure that our findings provide strong support for clinical decision making.

## Conclusion

Our findings demonstrated that a 2-week ketogenic diet intervention partially mitigated the metabolic phenotypes of MASLD, restored equilibrium in mitochondrial dynamics, and ameliorated mitochondrial dysfunctions in the livers of MASLD mice. As such, this study offers valuable insights into potential therapeutic strategies for MASLD by targeting mitochondrial homeostasis. There are some limitations of this study, such as the fact that this study only focused on the mitochondrial mechanism of β-OHB, and the lipid fraction of the ketogenic diet and its clinical use lacked detailed elaboration. In future studies, we will further focus on ah other ketone bodies and the clinical effects of the ketogenic diet.

## Supplementary information

Supplementary material

## Data Availability

The data supporting this article have been included as part of the Supplementary Information.
