# Supplementary material for: Ketogenic diet ameliorates MASLD via balancing mitochondrial dynamics and improving mitochondrial dysfunction
Source: Nutr Diabetes. 2025 Aug 25;15:37. doi: 10.1038/s41387-025-00391-w (PMC12375717; doi:10.1038/s41387-025-00391-w)
Supplement: Supplementary file 1 — Supplementary material [file 41387_2025_391_MOESM1_ESM.docx]

**Ketogenic Diet Ameliorates MASLD via Balancing Mitochondrial Dynamics and Improving Mitochondrial Dysfunction**

Yuehua You^1^, Hongbin Ni^1^, Qin Ma^1, 2^, Lincheng Jiang^1, 2^, Jingshu Cai^1^, Wenjun He^1^, Xiaojing Lin^1^, Kemeng Li^1^, Zhuyun Wang^1^, Weiyan Yan^1^, Xiaoqiu Xiao^1, §^ and Li Ma^1, §^

^1^ Department of Endocrinology, Sichuan-Chongqing Joint Key Laboratory of Metabolic Vascular Diseases, Chongqing Key Laboratory of Translational Medicine in Major Metabolic Diseases, The First Affiliated Hospital of Chongqing Medical University

^2^Department of Nutrition and Food Hygiene, School of Public Health and Management, Chongqing Medical University, Chongqing, 400016, China

^§^Corresponding authors: Li Ma, Department of Endocrinology, Sichuan-Chongqing Joint Key Laboratory of Metabolic Vascular Diseases, Chongqing Key Laboratory of Translational Medicine in Major Metabolic Diseases, The First Affiliated Hospital of Chongqing Medical University, No.1 Youyi Road, Yuzhong District, Chongqing, 400016, China. E-mail address: [malicheng2002@163.com](mailto:malicheng2002@163.com);

Xiaoqiu Xiao, Department of Endocrinology, Sichuan-Chongqing Joint Key Laboratory of Metabolic Vascular Diseases, Chongqing Key Laboratory of Translational Medicine in Major Metabolic Diseases, The First Affiliated Hospital of Chongqing Medical University, No.1 Youyi Road, Yuzhong District, Chongqing, 400016, China. E-mail address: 203679@cqmu.edu.cn

**Supplementary Table 1. Detailed composition of mouse diets used in this study**

| SD | gm | kcal |  | HFD | gm | kcal |  | KD | gm | kcal |
| --- | --- | --- | --- | --- | --- | --- | --- | --- | --- | --- |
| Protrin | 14.2% | 14.7% |  | protrin | 26.2% | 20% |  | protrin | 17.6% | 10.4% |
| Carbohydrate | 73.1% | 75.9% |  | Carbohydrate | 26.3% | 20% |  | Carbohydrate | 0.2% | 0.1% |
| Fat | 4% | 9.4% |  | Fat | 34.9% | 60% |  | Fat | 67.2% | 89.5% |
| Total kcal/gm | 3.85% | 100% |  | Total kcal/gm | 5.24% | 100% |  | Total kcal/gm | 6.76% | 100% |
| Casein,30Mesh | 140 | 560 |  | Casein,30Mesh | 200 | 800 |  | Casein,30Mesh | 100 | 400 |
| L-Cystine | 1.8 | 7.2 |  | L-Cystine | 3 | 12 |  | L-Cystine | 1.5 | 6 |
| Corn Starch | 495.692 | 1983 |  | Corn Starch | 0 | 0 |  | Corn Starch | 0 | 0 |
| Maltodextrin 10 | 125 | 500 |  | Maltodextrin 10 | 125 | 500 |  | Dextrose | 0 | 0 |
| Sucrose | 100 | 400 |  | Sucrose | 68.8 | 275.2 |  | Sucrose | 0 | 0 |
| Cellulose | 50 | 0 |  | Cellulose,BW200 | 50 | 0 |  | Cellulose,BW200 | 50 | 0 |
| Soybean Oil | 40 | 360 |  | Soybean Oil | 25 | 225 |  | Soybean Oil | 50 | 450 |
| T-Butylhydroquinone | 0.008 | 0 |  | Lard* | 245 | 2205 |  | Primex | 338 | 3042 |
| Mineral mix S10022M | 35 | 0 |  | Mineral mix S10026 | 10 | 0 |  | Mineral mix S1001 | 35 | 0 |
| Vitamin mix V10037 | 10 | 40 |  | Vitamin mix V10001 | 10 | 40 |  | Vitamin mix V10001C | 1 | 4 |
| Choline Bitartrate | 2.5 | 0 |  | Choline Bitartrate | 2 | 0 |  | Choline Bitartrate | 2 | 0 |
